# 不同类型肺结节的生长曲线分析

**DOI:** 10.3779/j.issn.1009-3419.2017.05.06

**Published:** 2017-05-20

**Authors:** 欣悦 王, 融诚 韩, 芳芳 郭, 欣菱 李, 文松 郑, 卿 王, 文静 宋, 铁链 于, 颖 王

**Affiliations:** 1 300052 天津，天津医科大学总医院医学影像科 Department of Radiology, Tianjin Medical University General Hospital, Tianjin 300052, China; 2 300052 天津，天津医科大学总医院医学病理科 Department of Pathology, Tianjin Medical University General Hospital, Tianjin 300052, China; 3 550002 贵阳 贵州省人民医院核医学科 Department of Nuclear medicine, Guizhou Provincial People's Hospitial, Guiyang 550002, China

**Keywords:** 肺结节, 计算机体层摄影, 体积, 三维体积分析, 体积生长曲线, Pulmonary nodules, Computed Tomography, Volume, Three-dimensional volumetric analysis, Volume growth curve

## Abstract

**背景与目的:**

计算机断层扫描（computed tomography, CT）随访评估肺内结节的生长特性是临床判断结节良恶性的常用策略。不同生物学行为的肺结节可能具有不同的生长速度和生长模式。本研究的目的是绘制不同类型肺结节的体积生长曲线，了解其生长方式，为判断结节性质并制定肺结节随访方案提供依据。

**方法:**

应用三维分析软件对111例接受2次及以上CT检查的肺结节（实性结节54例、亚实性结节57例）的影像资料进行回顾性分析。35例恶性及5例良性结节经病理或组织学确认，其余71例经两年随访无显著生长，经专家会诊确认为肺癌低危结节。所有结节按密度及性质分组：实性良性/低危结节、实性恶性结节、亚实性良性/低危结节、亚实性恶性结节。以随访间隔时间（d）为X轴，以随访结节的三维体积（mm^3^）和三维体积对数为Y轴，绘制体积线性及指数性生长曲线，由研究者主观观察曲线的形态。应用卡方检验比较不同性质肺结节的生长曲线的差异。

**结果:**

实性恶性结节中12例（66.7%）生长曲线快速上升，3例（16.7%）先平缓-后上升，2例（11.1%）缓慢上升，1例（5.56%）平直。亚实性恶性结节中8例（47.1%）呈快速上升型，4例（23.5%）缓慢上升，3例（17.6%）平直，2例（11.8%）为先下降-后上升型。实性良性/低危结节中5例（13.9%）呈下降型，17例（47.2%）平直，8例（21.6%）缓慢上升，6例（16.7%）呈波浪型。亚实性良性/低危结节中4例（10%）呈下降型，21例（52.5%）平直，9例（22.5%）缓慢上升，6例（15%）呈波浪型。良性/低危结节与恶性结节生长曲线分布存在显著性差异（*χ*^2^=42.4, *P* < 0.01）。

**结论:**

肺癌生长曲线具有异质性，快速上升是恶性肺结节的特征性生长曲线，但部分可在一定时期内表现为平直、缓慢上升甚至下降。缓慢生长不能排除肺癌可能，尤其是亚实性结节。

## Abstract (en)

**Background and objective:**

Background and objective Follow up by computed tomography (CT) and growth evaluation are routine methods for the differential diagnosis of indeterminate pulmonary nodules in clinical practice. Pulmonary nodules with diverse biological behaviors may show different growth patterns and velocities. The aim of this study is to identify the volume growth curve of both benign and malignant pulmonary nodules. This work also intends to determine these nodules' growth patterns and provide evidence for the establishment of a follow-up strategy.

**Methods:**

The CT data of 111 pulmonary nodules (54 solid, 57 subsolid) were retrospectively evaluated using 3D volumetric software. All of these nodules have been followed up at least twice. Of these nodules, 35 were confirmed as lung cancers, whereas 5 were confirmed as benign by pathology or histology. Moreover, 71 nodules showed no growth in more than 2 years. Stable nodules were defined as low-risk nodules, as confirmed by reevaluation from experts. On the basis of their densities and diameters, the nodules were classified into four types: benign/low-risk solid nodules, malignant solid nodules (diameter ≤1 cm and >1 cm), benign/lowrisk subsolid nodules, and malignant subsolid nodules (diameter ≤1 cm and >1 cm). The follow-up interval time (d) were plotted on the x-axis, and the nodules' volume (mm^3^) and logarithmic volume were plotted on the y-axis. Two radiologists subjectively determined the type of growth curve. Chi-square test was performed to compare the growth curves of benign/low-risk and malignant nodules.

**Results:**

Of 18 solid cancers, 12 cases (66%) were found with steep ascendant growth curves. Those of 3 cases (16.7%) were flat ascendant, 2 cases (11.1%) slowly ascendant, and 1 (5.56%) case flat. Of 17 subsolid cancers, 8 cases (47.1%) manifested steep ascendant growth curves. Those of 4 cases (23.5%) were slowly ascendant, 3 (17.6%) flat, and 2 (11.8%) descendant–ascendant. Of 36 benign/low-risk solid nodules, 5 cases (13.9%) manifested descendant growth curves, 17 cases (47.2%) flat, 8 cases (21.6%) slowly ascendant, and 6 cases (16.7%) undulate. Of 40 benign/low-risk subsolid nodules, 4 cases (10%) manifested descendant growth curves, 21 cases (52.5%) flat, 9 cases (22.5%) slowly ascendant, and 6 cases (15%) undulate. The distribution of growth curve types significantly differed between benign/low-risk and malignant nodules (*χ*^2^=42.4, *P* < 0.01).

**Conclusion:**

The growth curves of lung cancers are heterogeneous. A steep ascendant curve is the main type for lung cancer, with the exception of flat, slowly ascendant, or even descendant curve. A slowly ascendant curve cannot exclude the diagnosis of lung cancer, especially for subsolid nodules.

肺癌是世界上发病率和死亡率最高的恶性肿瘤^[1]^，其发病率在我国呈逐年快速增长趋势，在恶性肿瘤中居首位^[2]^。肺癌5年生存率T1期为48%（≤2 cm）和43%（2 cm-3 cm），T3期为27%，T4期伴胸膜转移仅为2%^[3]^。早期诊断、早期治疗是降低肺癌死亡率的重要措施。

计算机断层扫描（computed tomography, CT）在临床实践中的广泛应用使大量偶发肺结节被检出。绝大多数肺结节为良性病变，如何在众多结节病变中检出肺癌是临床的难题。随访评估其生长性是针对不能定性肺结节的常用策略。不同类型、不同性质的肺结节的生长速度和生长模式存在差异。基于指数生长模型^[4]^的体积倍增时间（volume doubling time, VDT）常用来量化肺结节生长速度。Hasegawa等^[5]^报道了恶性磨玻璃密度结节、部分实性结节与实性结节的平均VDT分别是813 d、457 d和149 d。然而，肺结节的生长速率受很多因素影响，其在特定时间段内的变化可能存在一定的变异。本次研究拟通过绘制不同类型及不同性质肺结节体积生长曲线观察肺结节生长方式，为判断结节性质并制定肺结节随访策略提供依据。

## 1 材料和方法

### 1.1 患者资料

回顾性分析2006年8月-2017年2月在天津医科大学总医院医学影像科行16或64排螺旋CT检查发现的肺结节。纳入标准：①首次CT检查结节大小为5 mm-3 cm；②随访次数至少2次，手术或穿刺获得病理的结节随访时间不少于1个月，随访无变化结节随访时间至少2年，术前或随访过程中未经临床干预；③排除急性肺炎或具有明显感染症状病例。良性结节及恶性结节经病理或组织学确认。

共纳入111例患者（男性49例，女性62例），年龄范围27岁-97岁，平均年龄（61.8±12.7）岁。恶性结节（35例）及良性结节（5例）均通过术后病理证实，其中肺间质纤维化1例，结核2例，错构瘤1例，不典型增生1例，原位腺癌2例，鳞癌5例，侵袭性腺癌28例。71例结节经2年及以上随访（730 d-3, 833 d，平均1, 054 d）体积无明显增长，因此类结节中包含亚实性结节，2年无著变不能作为良性的标准，经专家会诊确定为肺癌低危结节并与良性结节共同分析。

### 1.2 图像采集

所有检查均使用16或64排螺旋CT进行扫描，扫描范围自胸廓入口至肺底部，患者一次吸气后屏气完成全肺扫描，扫描方式：螺旋扫描；管电压：120 kv；管电流：300 mA；螺距：1.375:1；扫描层厚：5 mm；机架旋转一周时间：0.4 s；视野（field of view, FOV）：360 mm；图像矩阵：512×512。默认重建算法为标准算法，重建1.25 mm层厚轴位图像。

### 1.3 图像分析

结节的三维体积测量在GE AW4.6工作站上进行。由两位放射科医师分别进行两次测量，两次测量间隔时间超过1周。三维体积测量应用高级肺结节分析（advanced lung analysis, ALA）软件。具体操作步骤如下：选择入选患者的一组横断位图像（层厚1.25 mm），进入体积分析界面，选择目标结节最大截面积所在层面，根据医生对结节密度和位置的观察，选择结节相应的密度选项（实性、亚实性、磨玻璃密度）和位置选项（肺实质内、贴血管或贴胸膜）。ALA软件则对结节进行自动分割，并显示分割后的三维结节图像。在横断位、矢状位及冠状位三个方位的图像上观察结节的分割情况，对分割不满意的结节通过重新设定目标结节的中心提取位置、改变结节的周围提取范围（3 mm-30 mm）进行调整以达最佳分割效果。如果经调整后结节分割的区域与目标结节基本重合，则为分割满意，否则为分割不满意并从研究对象中剔除。ALA软件同时自动计算出分割后结节的体积V（mm^3^）。计算结节两次体积测量的平均体积（average volume, AV），AV=（第一次测量V+第二次测量V）/2。

所有结节根据密度及性质分组：A1：实性良性结节或肺癌低危结节；A2：实性恶性结节（A2-1：直径≤1 cm；A2-2：直径 > 1 cm）；B1组：亚实性良性结节或肺癌低危结节；B2组：亚实性恶性结节（B2-1：直径≤1 cm；B2-2：直径 > 1 cm）。

### 1.4 计算方法

以随访间隔时间（天）为X轴，其中第一次检查时间为0天；以随访CT检查结节的三维体积（mm^3^）及体积对数（lgAV）为Y轴，绘制结节的体积生长曲线图。图中每一条曲线代表一例肺结节的生长情况。采用体积对数的原因在于肺癌的生长模型包括指数模型，对数转换后更易于观察结节的生长特性。在标准（Y轴为体积）生长曲线中，曲线的斜率提示结节体积变化的幅度，生长缓慢的结节曲线斜率较小，生长快速的结节曲线斜率较大。对于多次随访的结节，其曲线在标准曲线中呈直线提示线性增长；在对数（Y轴为体积对数）生长曲线中呈直线代表指数型生长。

两位放射科医师对结节曲线进行分型。依据结节首次及末次的体积变化将结节生长曲线定义为上升、平稳、下降3型。基于曲线的形态及斜率，上升型曲线进一步细化为快速上升、缓慢上升、先平缓-后加速上升、先下降-后上升型。平稳型曲线进一步细分为平直及波浪型。

### 1.5 统计学分析

应用卡方检验评估不同性质肺结节的生长曲线类型是否存在差异。采用SPSS19.0软件完成统计分析，以*P* < 0.05认为差异有统计学意义。

## 2 结果

### 2.1 实性结节生长曲线

36例实性良性/低危结节中5例生长曲线为下降型，17例平直，6例呈波浪型，8例缓慢上升（图 1）。18例实性恶性结节中，直径≤1 cm结节7例，2例生长曲线呈快速上升，2例呈先平缓-后加速上升，1例平直（图 2），2例缓慢上升（图 3）。直径 > 1 cm结节11例，9例呈快速上升，2例呈缓慢上升（图 4）。

**1:**
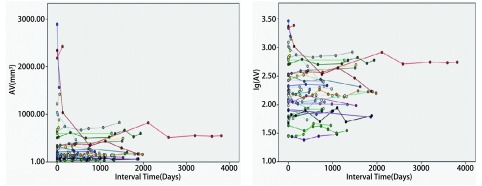
实性良性与低危结节体积及体积对数生长曲线 The volume and logarithm-volume growth curves of solid benign/low-risk nodules

**2:**
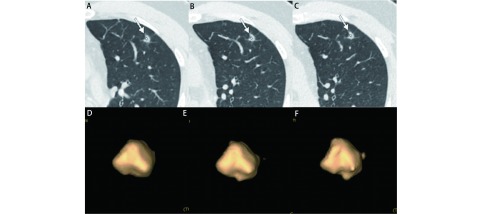
男，47岁，左上叶实性结节，直径约为1.0 cm，病理为浸润性腺癌。A、B：2013/03/12第一次CT检查，体积为211 mm^3^；C、D：2013/10/10检查，体积为235 mm^3^；E、F：2014/11/27检查，体积为267 mm^3^。在20个月的随访中，结节体积无明显增长，但术后病理诊断为腺癌。 Male, 47 years old, solid nodule in upper left lobe, diameter is 1.0 cm, pathology result was invasive adenocarcinoma. A, B: the first examination at 2013/03/12, the volume was 211 mm^3^; C, D: 2013/10/10, the volume was 235 mm^3^; E, F: 2014/11/27, the volume was 267 mm^3^. In more than 20 months of follow-up, nodule volume did not grew significantly, but pathology result was adenocarcinoma.

**3:**
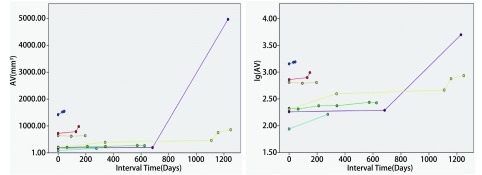
实性恶性结节体积及体积对数生长曲线（直径≤1 cm） The volume and logarithm-volume growth curves of solid malignant nodules (diameter ≤1 cm)

**4:**
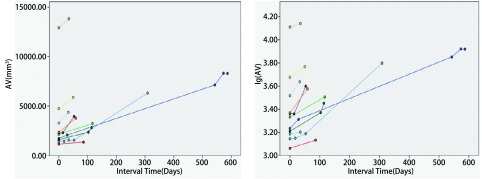
实性恶性结节体积及体积对数生长曲线（直径 > 1 cm） The volume and logarithm-volume growth curves of solid malignant nodules (diameter > 1 cm)

### 2.2 亚实性结节生长曲线

亚实性低危结节40例中，纯磨玻璃密度结节28例，部分实性结节12例。4例生长曲线呈下降型，21例平直，9例缓慢上升，6例呈小波浪型（图 5）。

**5:**
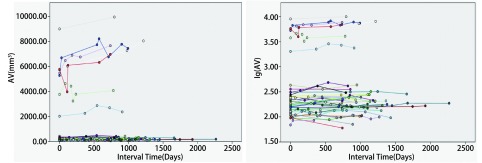
亚实性低危结节体积及体积对数生长曲线 The volume and logarithm-volume growth curves of subsolid low-risk nodules

亚实性恶性结节17例中，纯磨玻璃密度结节4例，部分实性结节13例。直径≤1 cm结节9例，4例结节曲线快速上升，3例缓慢上升（图 6），1例曲线平直，1例结节为先下降后上升趋势（图 7）。直径 > 1 cm结节8例，4例结节曲线呈快速上升趋势，1例结节缓慢上升，2例平直，1例结节为先下降后上升趋势（图 8）。

**6:**
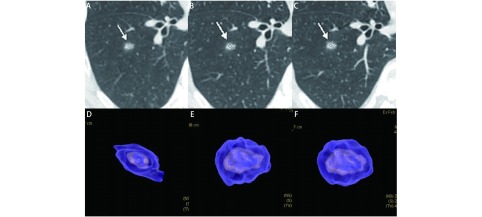
男，56岁，右下叶亚实性结节，直径约为0.9 cm，病理为浸润性腺癌，腺泡为主。A、B：2013/2/18第一次CT检查，体积为368 mm^3^；C、D：2013/5/3检查，体积为409 mm^3^；E、F：2014/2/19检查，体积为485 mm^3^。初次检查1年后亚实性结节增大缓慢。 Male, 56 years old, subsolid nodule in lower right lobe, diameter is 0.9 cm, pathology result was invasive adenocarcinoma; A, B: the first examination at 2013/2/18, the volume was 368 mm^3^; C, D: 2013/5/3, the volume was 409 mm^3^. E, F: 2014/2/19, the volume was 485mm^3^. In more than 1 year follow-up after initial inspection, the nodule grew slowly.

**7:**
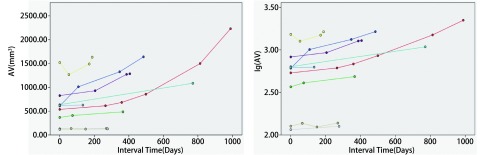
亚实性恶性结节体积及体积对数生长曲线（直径≤1 cm） The volume and logarithm-volume growth curves of subsolid malignant nodules (diameter ≤1 cm)

**8:**
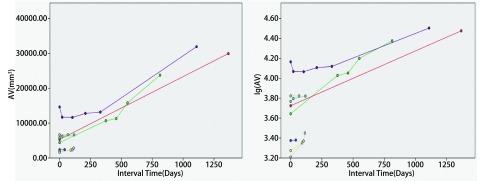
亚实性恶性结节体积及体积对数生长曲线（直径 > 1 cm） The volume and logarithm-volume growth curves of subsolid malignant nodules (diameter > 1 cm)

### 2.3 良性/低危结节与恶性结节生长曲线类型分析

恶性结节中88.6%（31/35）生长曲线在至少某一时段显示为上升型，11.4%（4/35）生长曲线呈水平型。实性良性/低危结节中22.3%（17/76）生长曲线为上升型，但均为缓慢上升，77.7%（59/76）的生长曲线为非上升型，包括平直、下降及波浪型（表 1）。良性/肺癌低危结节与恶性结节生长曲线类型分布存在显著性差异（*χ*^2^=42.4, *P* < 0.01）。

**1:** 111例不同性质肺结节的生长曲线类型 The growth curve types of 111 pulmonary nodules with different characteristics

Nodule charcteristics		Growth curve types								Total
		Descendant	Flat			Ascendant				
			Horizontal	Undulate		Slowly	Steep	Descendant-ascendant	Flat-ascendant	
Malignant	Solid ≤1 cm	0	1	0		2	2	0	2	7
	Solid > 1 cm	0	0	0		2	9	0	0	11
	Subsolid ≤1 cm	0	1	0		3	4	1	0	9
	Subsolid > 1cm	0	2	0		1	4	1	0	8
Benign and low-risk	Solid	5	17	6		8	0	0	0	36
	Subsolid	4	21	9		6	0	0	0	40

## 3 讨论

在临床实践中，不定性肺结节的CT随访对判断结节性质具有重要的临床意义。通过体积生长曲线的绘制，可以了解不同类型良恶性肺结节的生长趋势，有助于判断肺结节性质、制定不定性肺结节的随访方案、减少肺结节过度医疗。

目前存在的常用肿瘤生长模型包括指数生长模型、线性生长模型及Gompertzian生长模型^[4]^。指数模型的理论基础为恶性肿瘤细胞不受限制地异常增殖。线性模型认为肿瘤的体积随着时间延长呈相应比例增长，其基础在于部分临床观察的结果。Gompertzian生长模型假设肿瘤最初以指数增长方式生长，但随着肿瘤体积增大，其生长速度逐渐减缓^[6]^。

难以节制的生长是肺癌结节的特征表现。我们的研究发现大部分肺癌结节的生长曲线特征为上升型曲线，但上升的速率不同，部分可为缓慢增长，部分甚至可以在一段时间内保持平直甚至下降，意味着肿瘤生长过程中可发生无增长，不规律增长、增长加快及自发性衰退。我们的研究结果与Lindell等^[7]^研究者的肺癌生长曲线研究结论相似，提示目前存在的3种肺癌生长模型并不能完全解释肺癌的生长。其原因在于此3种模型均为理想情况下的生长假说，而在现实中肿瘤的生长受多种因素影响。首先，肿瘤生长取决于其所处环境，包括血供、营养及周围的空间限制。肿瘤结节的出血可能造成肿瘤突然变大，供血血管形成血栓可能导致肿瘤坏死和自然缩小。基本的营养、激素和化学因素也会影响肿瘤的增长速度。其次，肿瘤的体积并不完全由肿瘤细胞组成，还包括肿瘤基质、血液和其它非肿瘤成分。非肿瘤成分的比例对体积的增长具有影响。此外，同一肿瘤内肿瘤细胞可存在异质性，活跃度可能不同，导致生长速率出现变化。

实性及亚实性恶性结节所显示出的总体生长曲线速率也存在一定差异。在我们的研究中，通过生长曲线可以看出实性恶性结节中快速增长的比例较亚实性恶性结节高，亚实性结节较实性结节生长趋势平缓。这与已有的研究结果一致。既往研究^[8]^显示多数实性恶性结节生长较快，体积倍增时间在20 d-400 d之间；部分实性结节与纯磨玻璃密度结节生长较慢，平均体积倍增时间分别为（276.9±155.9）d和（628.5±404.2）d。其生物学基础在于不同密度结节的病理存在差异。恶性磨玻璃密度结节病理常为原位或微侵袭性腺癌，而随着实性成分的增加，侵袭性腺癌的比例增加。

结节大小与恶性风险度明确相关，同时也是制定结节管理的因素^[9]^。在恶性结节中，小结节生长曲线呈快速生长的比例较直径 > 1 cm结节小。即使两组均呈快速增长，恶性小结节较大结节生长趋势仍显平缓，且小结节均在随访后期开始快速增长。所以对于小结节，即使早期未表现为快速增长，仍不能排除其恶性可能性。

良性/肺癌低危结节生长曲线基本不表现为快速增长，但可表现为斜率较低的上升型曲线，与部分恶性结节的生长曲线具有一定的重叠。因此，在一段时间内结节快速增长可以作为恶性结节的阳性诊断标准，但平缓增长甚至体积下降不能作为阴性诊断标准。本研究中，2例实性恶性小结节表现为第一次随访无明显增长，但第二次随访显著增长，2例亚实性恶性结节生长曲线呈先下降后上升趋势。如果单纯依靠第一次随访的结果做出诊断，会造成假阴性的出现。我们认为肺癌结节在一定时间内平缓增长可能和结节的侵袭性较小或处于相对生长静止期有关。肺癌结节体积下降的原因可能为结节内发生纤维化导致体积减小或周围浸润性炎症消失所致。针对此现象，我们建议在临床实践中，应该延长随访时间或结合结节的其他临床及影像特性综合判断。

本研究也有一定的局限性，首先，病例的选择具有偏倚，临床上进行随访的肺结节多为较小且不具有典型表现的肺结节，因此纳入的恶性结节病例更倾向于包含较多相对惰性生长的肺癌，并不能代表肺癌全体。但本文的重点在于提出肺癌结节可以表现为不同形态的生长曲线，而不是描述肺癌总体的生长特性。其次，由于临床实践中肺结节随访患者失访率较高，我们采用的是回顾性研究，和前瞻性队列研究相比诊断效能不足。

总之，对不定性肺结节来说，生长较快的结节可初步判定为恶性结节，长期随访生长趋势平缓可初步认定为良性，但并不排除恶性的可能，部分恶性结节尤其是小结节和亚实性结节在一定时间内可表现为缓慢生长。

## References

[1] Torre LA, Bray F, Siegel RL (2015). Global cancer statistics, 2012. CA Cancer J Clin.

[2] Chen W, Zheng R, Zeng H (2015). Epidemiology of lung cancer in China. Thorac Cancer.

[3] Rami-Porta R, Ball D, Crowley J (2007). The IASLC Lung Cancer Staging Project: proposals for the revision of the T descriptors in the forthcoming (seventh) edition of the TNM classification for lung cancer. J Thorac Oncol.

[4] Brú A, Albertos S, Luis SJ (2003). The universal dynamics of tumor growth. Biophys J.

[5] Hasegawa M, Sone S, Takashima S (2000). Growth rate of small lung cancers detected on mass CT screening. Br J Radiol.

[6] Castro MA, Klamt F, Grieneisen VA (2003). Gompertzian growth pattern correlated with phenotypic organization of colon carcinoma, malignant glioma and non-small cell lung carcinoma cell lines. Cell Prolif.

[7] Lindell RM, Hartman TE, Swensen SJ (2009). 5-year lung cancer screening experience: growth curves of 18 lung cancers compared to histologic type, CT attenuation, stage, survival, and size. Chest.

[8] Oda S, Awai K, Murao K (2011). Volume-doubling time of pulmonary nodules with ground glass opacity at multidetector CT: Assessment with computer-aided three-dimensional volumetry. Acad Radiol.

[9] MacMahon H, Naidich DP, Goo JM (2017). Guidelines for management of incidental pulmonary nodules detected on CT images: From the Fleischner Society 2017. Radiology.

